# Obeticholic acid attenuates complete freund’s adjuvant-induced arthritis in rats through FXR-mediated modulation of TGF-β/TAK1/MKK3/p38 MAPK signaling, inflammation, and oxidative stress

**DOI:** 10.1007/s10787-026-02389-x

**Published:** 2026-09-18

**Authors:** Mohamed Magdy Senna, Nesma A. Shiha, Hala F. Zaki, Laila A. A. Ramadan, Aya M. Mustafa

**Affiliations:** 1 Department of Pharmacology and Toxicology, Faculty of Pharmacy, Egyptian Russian University, Badr City, Cairo, 11829 Egypt; 2 Department of Pharmacology and Toxicology, Faculty of Pharmacy, Cairo University, Cairo, Egypt

**Keywords:** Rheumatoid arthritis, Obeticholic acid, Farnesoid X receptor, TGF-β/TAK1/p38 MAPK pathway, Oxidative stress

## Abstract

Rheumatoid arthritis is a chronic autoimmune inflammatory disorder characterized by synovial inflammation, oxidative stress, cartilage destruction, and progressive joint damage. Farnesoid X receptor activation has recently emerged as a potential regulator of immune and inflammatory responses. This study investigated the effect of obeticholic acid (OCA), a potent farnesoid X receptor agonist, in a complete Freund’s adjuvant-induced arthritis model in rats, with emphasis on the TGF-β/TAK1/MKK3/p38 MAPK signaling pathway, inflammatory mediators, and oxidative stress.

Arthritis was induced in male Wistar rats by subplantar injection of complete Freund’s adjuvant on days 0 and 7. Rats were allocated into nine groups: normal control, OCA control, arthritic control, arthritic rats treated with OCA at 2.5 or 5 or 10 mg/kg, methotrexate-treated arthritic rats, F44-A13-treated arthritic rats, and arthritic rats co-treated with F44-A13 and OCA. Treatments were administered orally from day 8 for 14 consecutive days. Clinical arthritis severity, paw edema, pain behavior, body weight, immune organ weight, serological markers, inflammatory cytokines, oxidative stress markers, gene expression, and histopathological changes were evaluated.

Obeticholic acid significantly ameliorated arthritic manifestations, as evidenced by reduced arthritis score, paw swelling, pain behavior, spleen and thymus weight, and improved body weight. It also decreased serum rheumatoid factor, C-reactive protein, and anti-cyclic citrullinated peptide levels. Obeticholic acid treatment was associated with restoration of FXR expression and downregulation of TGF-β, TGF-βR1, TAK1, MKK3, and p38 MAPK expression, accompanied by suppression of NF-κB, TNF-α, IFN-γ, IL-1β, and IL-6. In parallel, obeticholic acid enhanced antioxidant defenses by increasing Nrf2, superoxide dismutase, and catalase while reducing malondialdehyde levels. Histopathological assessment confirmed attenuation of synovial inflammation, cartilage damage, and proteoglycan loss. Co-administration of the FXR antagonist F44-A13 markedly blunted the protective effects of obeticholic acid.

These findings indicate that OCA attenuates CFA-induced experimental arthritis and that these effects are associated with FXR-related changes in TGF-β/TAK1/MKK3/p38 MAPK gene expression, inflammatory mediators, and oxidative stress.

**Graphical abstract:**

**Figure:**
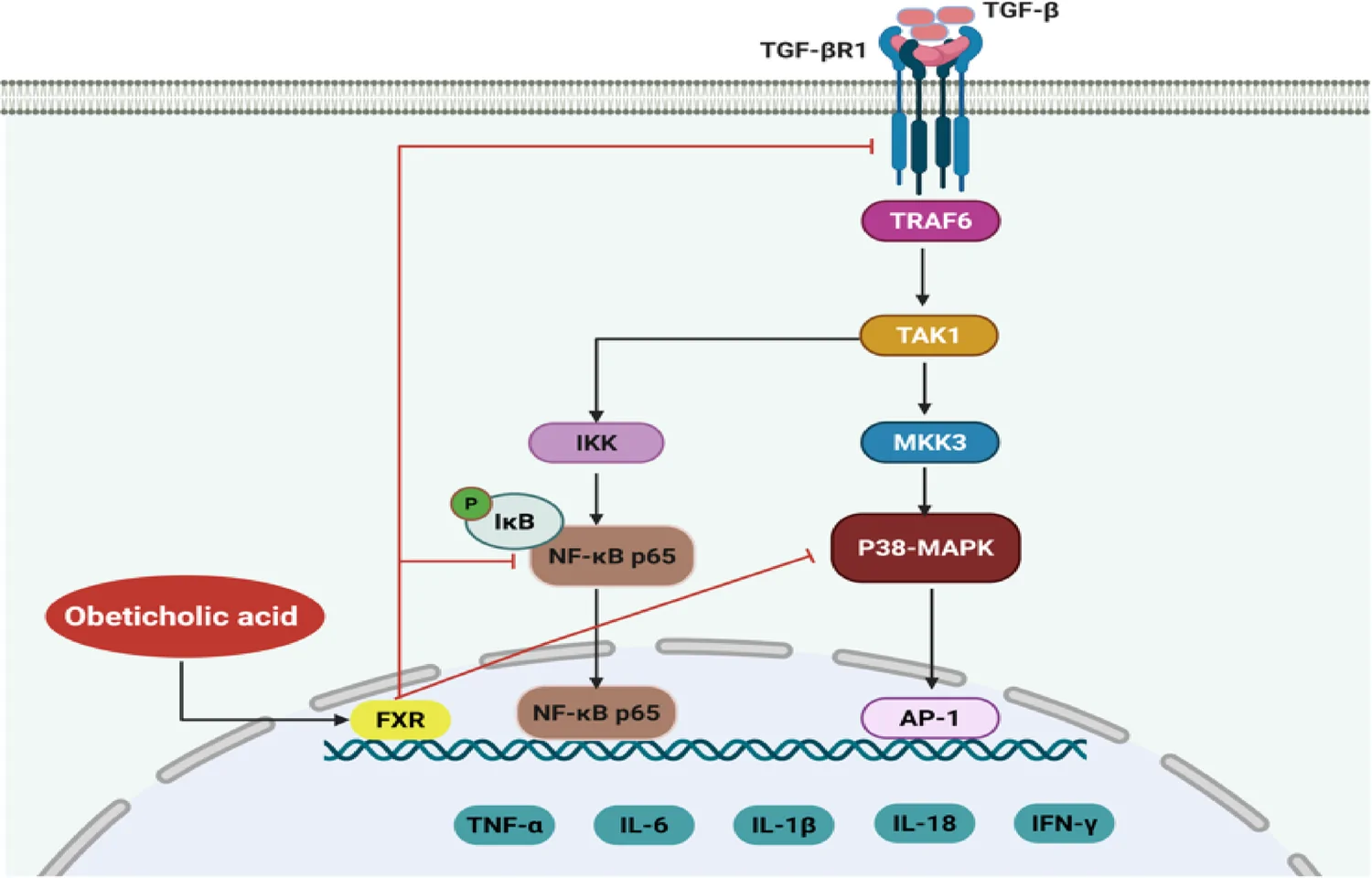

## Introduction

Rheumatoid arthritis (RA) is a systemic chronic autoimmune disease of noticeable progressive synovial hyperplasia and inflammation, joint deformity, and extra-synovial manifestations, as well as significant deterioration in quality of life due to chronic pain and functional impairment, driven by immune dysregulation and loss of self-tolerance (Radu and Bungau 2021; Karami et al. 2020; Rindfleisch and Muller 2005). In general, RA affects approximately 1% of the global population (Finckh et al. 2022).

To elucidate the underlying pathogenic mechanisms of RA and assess potential therapeutic interventions, several experimental models have been established. The Complete Freund's adjuvant (CFA)-induced arthritis (AIA) model is a widely used experimental model that closely mimics key features of RA, including bone erosion, cartilage destruction, and synovial hyperplasia (Choudhary et al. 2018).

Accumulating evidence highlights the involvement of the farnesoid X receptor (FXR), a bile acid-activated nuclear receptor, in modulating immune regulation and inflammatory responses (Anderson and Gayer 2021). FXR has been particularly implicated in the pathogenesis of inflammatory diseases affecting the gut, liver, and lungs, as well as autoimmune conditions such as lupus, autoimmune encephalitis, and multiple sclerosis, as previously demonstrated (Belka et al. 2024; Fu et al. 2022; Fang et al. 2021; Wang 2025; Jia et al. 2021). FXR is expressed across a range of immune cells and plays a pivotal role in regulating immune cell differentiation and cytokine secretion (Li et al. 2024). Notably, FXR activation in macrophages suppresses nuclear factor-kappa B (NF-κB) signaling, thereby decreasing the production of pro-inflammatory mediators (Huang et al. 2024). Recent findings suggest a mechanistic link between FXR activation and reduced inflammation through crosstalk with the transforming growth factor-β receptor (TGF-βR1) and transforming growth factor-β-activated kinase 1 (TAK1) (Li et al. 2020), which operates as a master regulator, integrating upstream signals and coordinating the activation of downstream pathways involving mitogen-activated protein kinase kinase (MKK), p38 mitogen-activated protein kinase (MAPK), and inhibitor of kappa B kinase (IKK) pathways (Touny et al. 2024).

In addition to immune dysregulation, oxidative stress has emerged as a critical contributor to RA pathogenesis. Reactive oxygen species (ROS) are significantly elevated in the synovial fluid of RA patients and promote abnormal proliferation and activation of fibroblast-like synoviocytes, thereby exacerbating synovial inflammation and joint destruction (Jing et al. 2023).

Obeticholic acid (OCA), a potent semi-synthetic bile acid derivative that functions as an agonist of the FXR, was initially developed for the treatment of primary biliary cholangitis (Buechter et al. 2025). However, it garnered significant attention for its broader therapeutic potential in the management of autoimmune and inflammatory diseases (Gadaleta et al. 2011a; Ho and Steinman 2016; Fei et al. 2019). By activating FXR, OCA modulates the expression of genes involved in immune regulation, thereby exerting anti-inflammatory and immunomodulatory effects (Abenavoli et al. 2018). These properties make OCA a promising candidate for the treatment of chronic inflammatory conditions.

Based on the aforementioned data, our study aimed to study the possible protective efficacy of OCA against CFA-induced arthritis in rats. Moreover, the aim was extended to elucidate the potential mechanisms involved in these effects, with a focus on the FXR/TGF-β/TAK1/MKK/p38 MAPK signaling cascade. To strengthen data reliability, Methotrexate (MTX), a well-established standard anti-arthritic drug, was used as a reference control for comparative evaluation (Kamel et al. 2019) as well as, F44-A13, a selective FXR antagonist, was used (Dou et al. 2024).

## Materials and methods

### Ethics statement

This study adheres to the ARRIVE guidelines and the National Institutes of Health Guide for the Care and Use of Laboratory Animals (NIH Publication No. 85–23, amended 2011). The Ethics Research Committee of the Faculty of Pharmacy, Cairo University (Cairo, Egypt) accepted the study (Permit number 3686). During the trial, every conceivable effort was made to mitigate animal pain.

### Animals

This research employed male Wistar rats weighing 180–200 g. All animals were obtained from El-Nile Company for Pharmaceutical and Chemical Industries (Cairo, Egypt) and acclimatized for one week before the experiment. The rats were housed under controlled conditions (12 h light/dark cycle, 60 ± 10% humidity, 23 ± 2 °C). They were granted unrestricted access to water and ad libitum access to normal chow.

### CFA-induced arthritis

Arthritis was induced by injecting 100 μL of Complete Freund's Adjuvant (CFA; heat-killed Mycobacterium tuberculosis in mineral oil; Sigma-Aldrich, MO, USA) into the right hind paw. A booster dose of 100 μL CFA was administered into the same paw after seven days to enhance the arthritic response (Saad et al. 2019).

### Assessment of anti-arthritic activity

Arthritis severity in the CFA-injected right paw was scored on a scale of 0–4: 0 = no swelling; 1 = mild swelling; 2 = moderate swelling; 3 = severe swelling; and 4 = whole-paw swelling. To minimize assessment bias, animals were identified using coded labels, and the order of evaluation was randomized at each time point. Pain behavior was evaluated by measuring paw withdrawal latency to ankle joint compression using a Randall-Selitto device. Paw volume change was determined using a plethysmometer (Ugo Basile 7140) and expressed in milliliters. Body weight was monitored throughout the study. All behavioral and clinical measurements were performed under standardized conditions by blinded assessors on days 1, 5, 8, 10, 14, 17, 19, 21.

### Experimental design

**Fig. 1:**
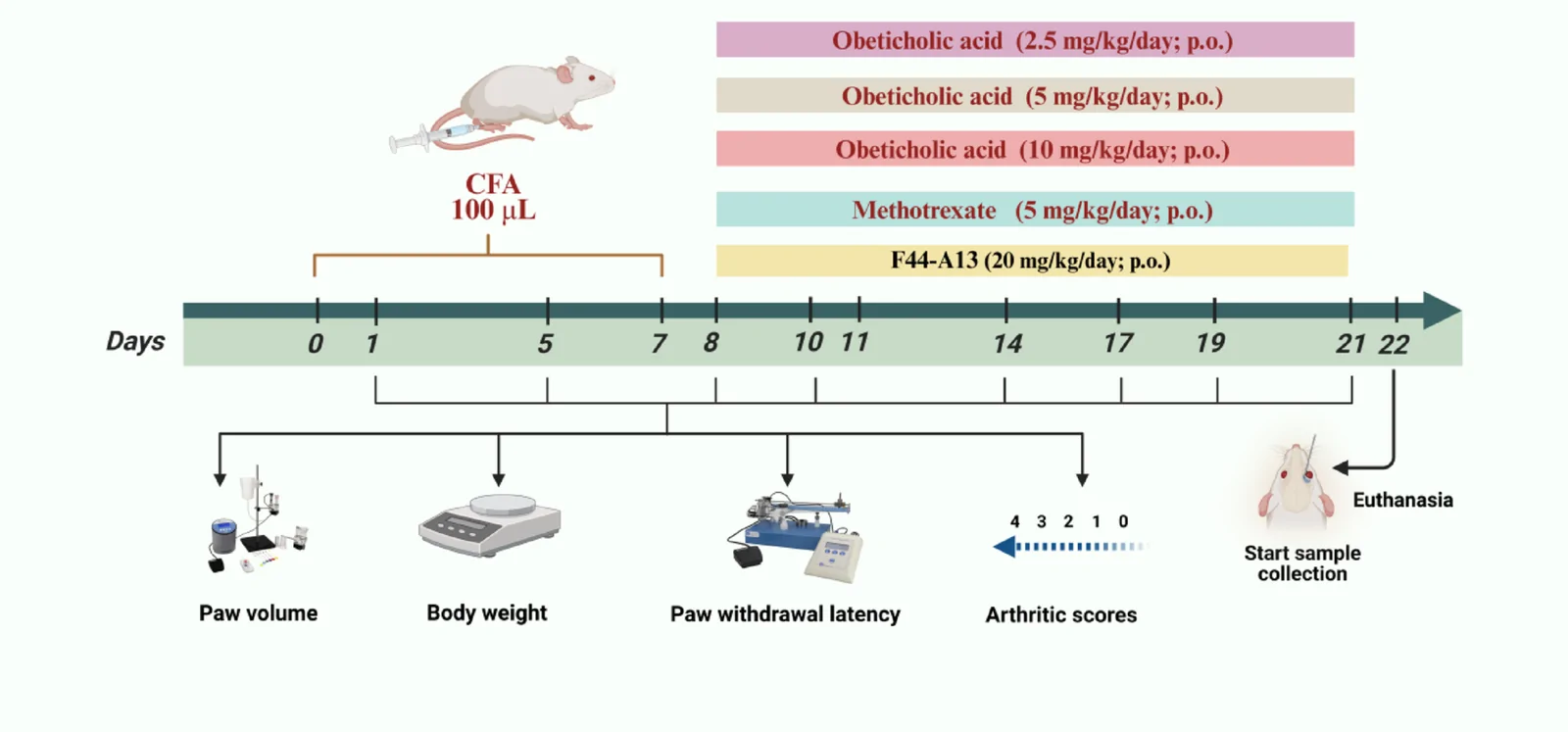
Schematic illustration for the experimental timeline, including behavioral assessments and treatment administration

Rats were randomly assigned to nine groups (n = 12 /group). Group I received dimethyl sulfoxide (DMSO) (0.2 ml/kg/day; p.o.; Thermo Fisher, USA) and served as the normal control, while Group II received OCA (10 mg/kg/day, p.o.; Sigma-Aldrich, MO, USA) (Comeglio et al. 2019). Groups III-VIII were immunized with CFA at days 0 and 7 (100 μl; into the right hind paw) (Saad et al. 2019), whereas group III served as the CFA arthritic group, group IV received OCA (2.5 mg/kg; p.o.), group V received OCA (5 mg/kg; p.o.) (Úbeda et al. 2016), group VI received OCA (10 mg/kg; p.o.) (Comeglio et al. 2019), group VII received MTX (5 mg/kg; p.o.) (Tamizhmozhi and Nagavalli 2016). group VIII received F44-A13 (20 mg/kg/day; p.o.; MedChemExpress, NJ, USA) (Dou et al. 2024), and group IX received F44-A13 (20 mg/kg/day; p.o.) and OCA (10 mg/kg; p.o.). OCA, MTX and F44-A13 were administered daily starting on day 8 for 14 consecutive days (Dou et al. 2024; Úbeda et al. 2016). OCA and F44-A13 were dissolved in DMSO. On day 22, rats were anesthetized with thiopental sodium (50 mg/kg, i.p.) (Ahmed et al. 2024), and blood samples were collected from the retro-orbital sinus. Animals were then sacrificed by cervical dislocation. The spleen and thymus were excised, rinsed with saline, and weighed, while hind paws were harvested for subsequent biochemical and histopathological assessment. Figure 1 illustrates the experimental timeline, including behavioral assessments and treatment administration.

### Biochemical parameters

#### Assessment of serological markers of RA

Serum inflammatory markers were measured using ELISA kits (MyBioSource, CA, USA): rheumatoid factor (RF; Cat# MBS702417), C-reactive protein (CRP; Cat# MBS2018890), and anti-cyclic citrullinated peptide (anti-CCP; Cat# MBS762429). RF was expressed in mIU/mL, while CRP and anti-CCP were expressed in ng/mL.

#### ELISA assay of IFN-γ, Nrf2, IL-6, IL-1β, TNF-α and NF-κB

IFN-γ (Cat# MBS766197) and Nrf2 (Cat# MBS752046) were measured in paw tissue using ELISA kits from MyBioSource (CA, USA), NF-κB p65 (Cat# CSB-E13148r) and TNF-α (Cat# CSB-E11987r) using a Cusabio kit (Wuhan, PRC), IL-1β (Cat# E-EL-R0012) and IL-6 (Cat# E-EL-R0015) using an Elabscience kit (Texas, USA). All assays were performed according to the manufacturers' protocols, and results were normalized to the measured protein content in the sample and expressed as pg/mg protein for IFN-γ, IL-6, IL-1β, NF-κB, and TNF-α, and as ng/mg protein for Nrf2.

##### Colorimetric assay of oxidative stress biomarkers

Superoxide dismutase (SOD) and catalase (CAT) activities, as well as malondialdehyde (MDA) levels, were determined in paw tissue using colorimetric assay kits (Biodiagnostic, Cairo, Egypt) in 10% (w/v) tissue homogenates. Results were expressed as U/g protein for SOD, U/mg protein for CAT, and nmol/g protein for MDA.

##### Quantitative RT-PCR

mRNA expression of FXR, TGF-β, TGF-βR1, TAK1, MKK3, and p38 MAPK was assessed by quantitative RT-PCR. Total RNA was extracted from paw tissue using the RNeasy Mini Kit (Qiagen), and RNA purity was evaluated spectrophotometrically by measuring the A260/A280 ratio. cDNA was synthesized from 1 μg of total RNA using the Reverse Transcription System (Promega) according to the manufacturer's instructions. Quantitative RT-PCR was carried out using SYBR Green Master Mix (Applied Biosystems) in a total reaction volume of 25 μL, containing 5 μL cDNA, 12.5 μL SYBR Green Master Mix, 5.5 μL RNase-free water, and 2 μL of each primer (Table 1). The amplification protocol consisted of 40 cycles of denaturation at 95 °C for 15 s, annealing at 60 °C for 60 s, and extension at 72 °C for 60 s.

**Table 1:** Primer sequences used for qPCR

mRNA species	Accession number	Primer sequence (5´–3´)
FXR	NM_001439627	F: CCAGGTCTCACTCAAGAATC R: GTCCCTAGCTCAGTTGACAA
TGF-β	NM_021578	F: CCTGCACAGCTCCAGGCACC R: TGCTCCACCTTGGGCTTGCG
TGF-βR1	NM_012775	F: GAAATCGCTCGACGCTGTTC R: GAACAACCGGCCTCCAAAAC
TAK1	NM_001107920	F: ATGCAGCCCAAAGCTCTCAT R: GCCCCGTTTAGGCTTGGTAT
MKK3	NM_001100674	F: TTACATGGCCCCTGAGAGGA R: CTTGGGACAGCTAGTTGCGA
p38 MAPK	NM_031020	F: TCGGCACACTGATGACGAAA R: TCATGGCTTGGCATCCTGTT
GAPDH	NM_017008	F: AATGGGCAGCCGTTAGGAAA R: GCGCCCAATACGACCAAATC

Primer specificity was validated by the presence of a single amplification product and confirmed by melt curve analysis, which showed a single sharp peak for each primer pair, indicating the absence of nonspecific products or primer-dimer formation. Amplification efficiency for each target and the reference gene was evaluated using standard curves generated from serial cDNA dilutions. It was found to be within the acceptable range for relative quantification. The stability of GAPDH expression was also verified across all experimental groups, and GAPDH was therefore used as the internal reference gene for normalization. Relative mRNA expression was calculated using the 2^-ΔΔCt method.

### Histopathological examination

Hind paws were dissected and fixed in 10% neutral-buffered formalin for 72 h, with the fixative renewed daily. After trimming, tissues were dehydrated through a graded ethanol series, cleared in xylene, and embedded in synthetic wax (Paraplast tissue-embedding medium). Sagittal sections of the paws (4–6 μm) were cut using a rotary microtome and stained with hematoxylin and eosin (H&E) for general histopathological evaluation and with safranin-O stain for assessment of cartilage matrix proteoglycan content under a light microscope (Bancroft and Gamble 2008). Histopathological and Mankin score assessments were performed in a blinded manner by a trained observer who was unaware of treatment allocation at the time of assessment. Cartilage damage was semiquantitatively evaluated using the Mankin scoring system, based on cartilage surface damage, loss of cellularity, loss of matrix staining, and loss of tidemark integrity (Mankin, et al. 1971). Although the Mankin scoring system was originally developed for osteoarthritic cartilage lesions, it was applied in the present CFA-induced arthritis model because it provides a semiquantitative assessment of the main structural and matrix-related features of cartilage damage, including surface irregularity, chondrocyte loss, proteoglycan depletion, and tidemark disruption. These changes are relevant to arthritic joint degeneration and can be reliably demonstrated in safranin-O-stained sections, making Mankin scoring suitable for evaluating cartilage injury in this experimental setting.

### Statistical analysis

Data are presented as mean ± SD. A one-way ANOVA followed by Tukey's multiple-comparison test was used for all parameters. At the same time, arthritic score, paw withdrawal latency, paw volume, and body weight were analyzed using two-way ANOVA with Bonferroni's post hoc test. To evaluate the data's adherence to normal distribution, the Shapiro–Wilk test was performed, and all statistical analyses were conducted using GraphPad Prism version 9 (GraphPad Software, Inc., San Diego, CA), with *p* < 0.05 considered statistically significant.

## Results

### Effect of obeticholic acid on arthritic scoring in CFA-induced arthritic rats

**Fig. 2:**
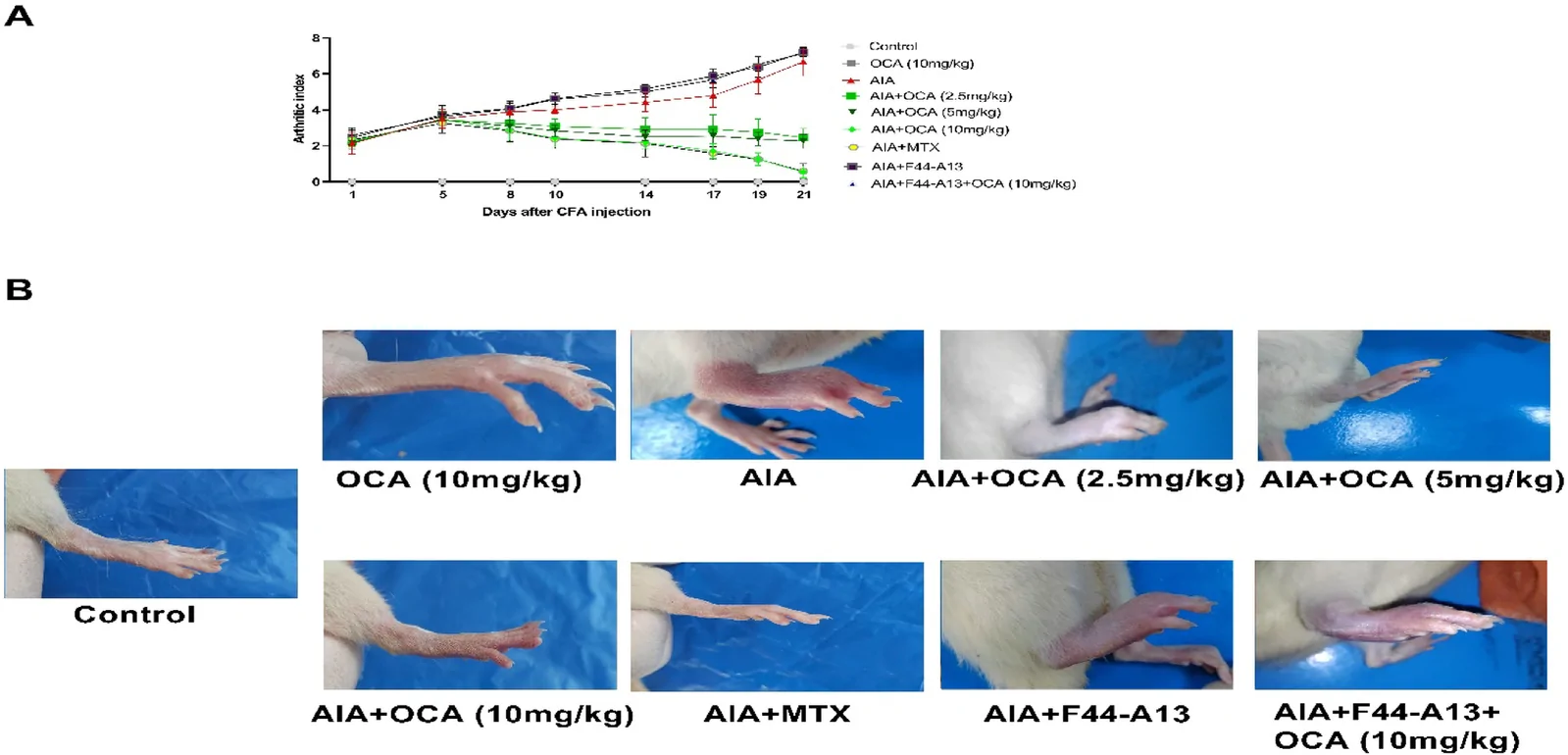
Effect of OCA on CFA-induced abnormalities in arthritic score. **A** Arthritic score and **B** Photographs of rats' hind paws. Data are expressed as mean ± SD of 12 rats per group, using two-way ANOVA and Bonferroni's post-hoc test; OCA: obeticholic acid; AIA: adjuvant-induced arthritis; MTX: methotrexate

Inflammation of the joint was assessed by determining the arthritic index as summarized in Fig. 2. There was a notable increase in the arthritic index in the CFA group, which progressively worsened over time. The severity of arthritis signs was exacerbated after the second immunization and remained elevated until the end of the experiment compared with control rats. Otherwise, after initiation of treatment with low-doses (2.5 and 5 mg/kg) and high-dose (10 mg/kg) of OCA on day 8, the arthritic index recovered relative to the arthritic rats, approaching near-normal values by the end of the treatment protocol. The effect demonstrated by OCA treatment was blunted by simultaneous administration of F44-A13.

### Effect of obeticholic acid on paw withdrawal latency and paw volume

**Fig. 3:**
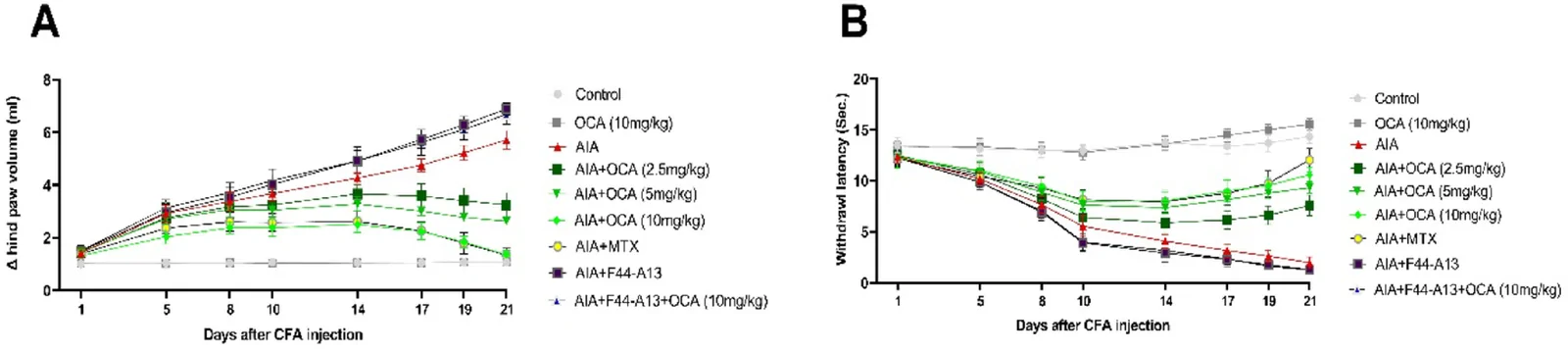
Effect of OCA on CFA-induced alterations in **A** Change in paw volume and **B** Paw withdrawal latency. Data are expressed as mean ± SD of 12 rats per group, using two-way ANOVA and Bonferroni's post-hoc test; OCA: obeticholic acid; AIA: adjuvant-induced arthritis; MTX: methotrexate

To explore the effect of OCA on inflammation and inflammatory pain, hind paw volume and behavioral assessments were measured during the experimental period after the start of CFA injection, as shown in Fig. 3. CFA injection increased the paw volume and reduced paw withdrawal latency in relation to the control group. Treatment with low doses (2.5 and 5 mg/kg) and high-dose (10 mg/kg) OCA showed a gradual improvement in these alterations, compared to the insult group, which approached near-control values for paw volume by the end of the protocol period. On the other hand, the results showed that F44-A13 reversed the beneficial effect of OCA when co-administered.

### Effect of obeticholic acid on body weight in CFA-induced arthritic rats

**Fig. 4:**
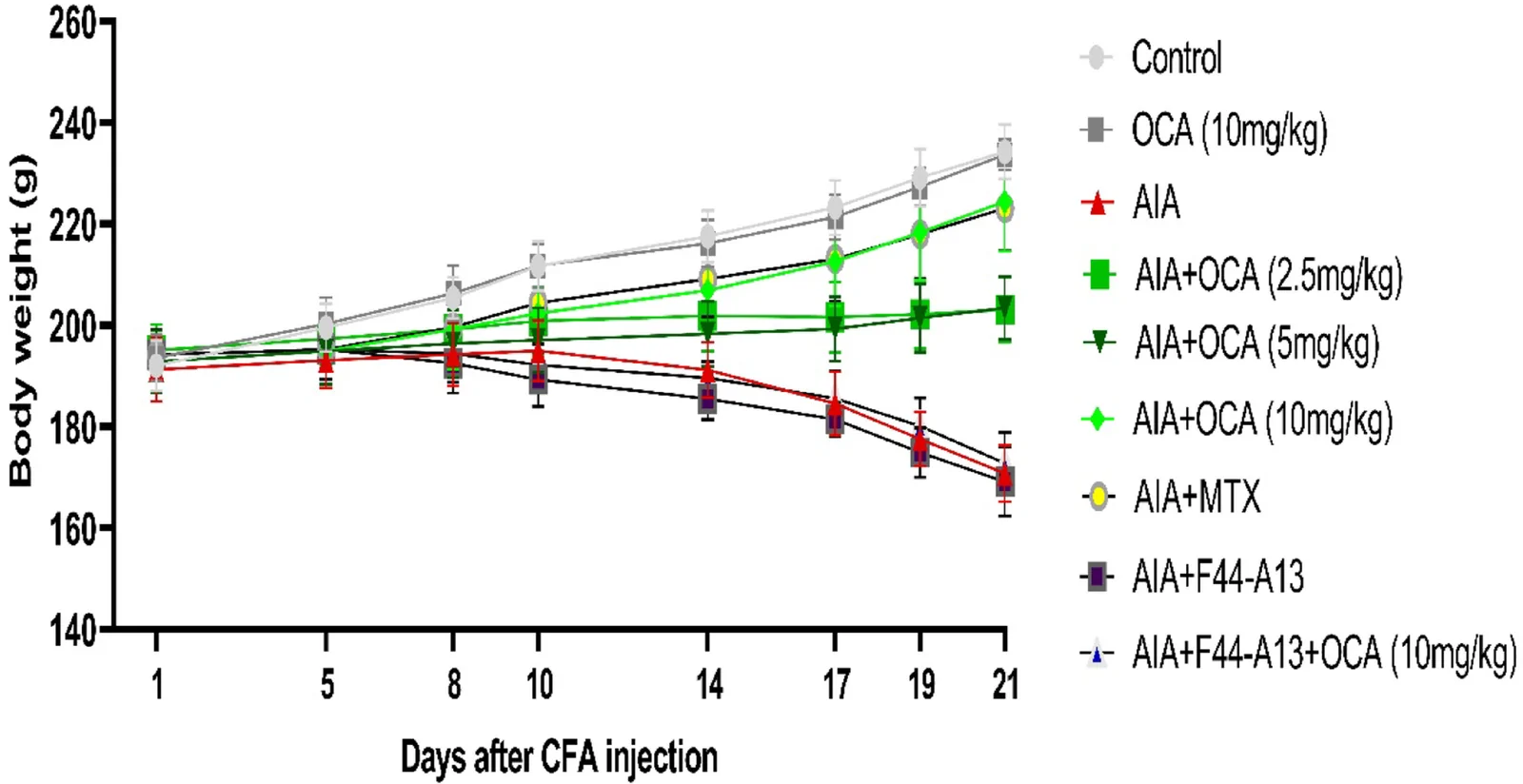
Effect of OCA on CFA-induced body weight change. Data are expressed as mean ± SD of 12 rats per group, using two-way ANOVA and Bonferroni's post-hoc test; OCA: obeticholic acid; AIA: adjuvant-induced arthritis; MTX: methotrexate

Body weight was measured at different time points, as shown in Fig. 4. There was a noticeable reduction in body weight in the CFA-injected group compared to the control group of rats. In contrast, body weight began to improve after the start of OCA treatments at both low and high doses, compared with the CFA group, and continued to improve through the end of the treatment period. Conversely, body weight declined with F44-A13 administration compared to the OCA group.

### Effect of obeticholic acid on thymus and spleen weight in CFA-induced arthritic rats

**Fig. 5:**
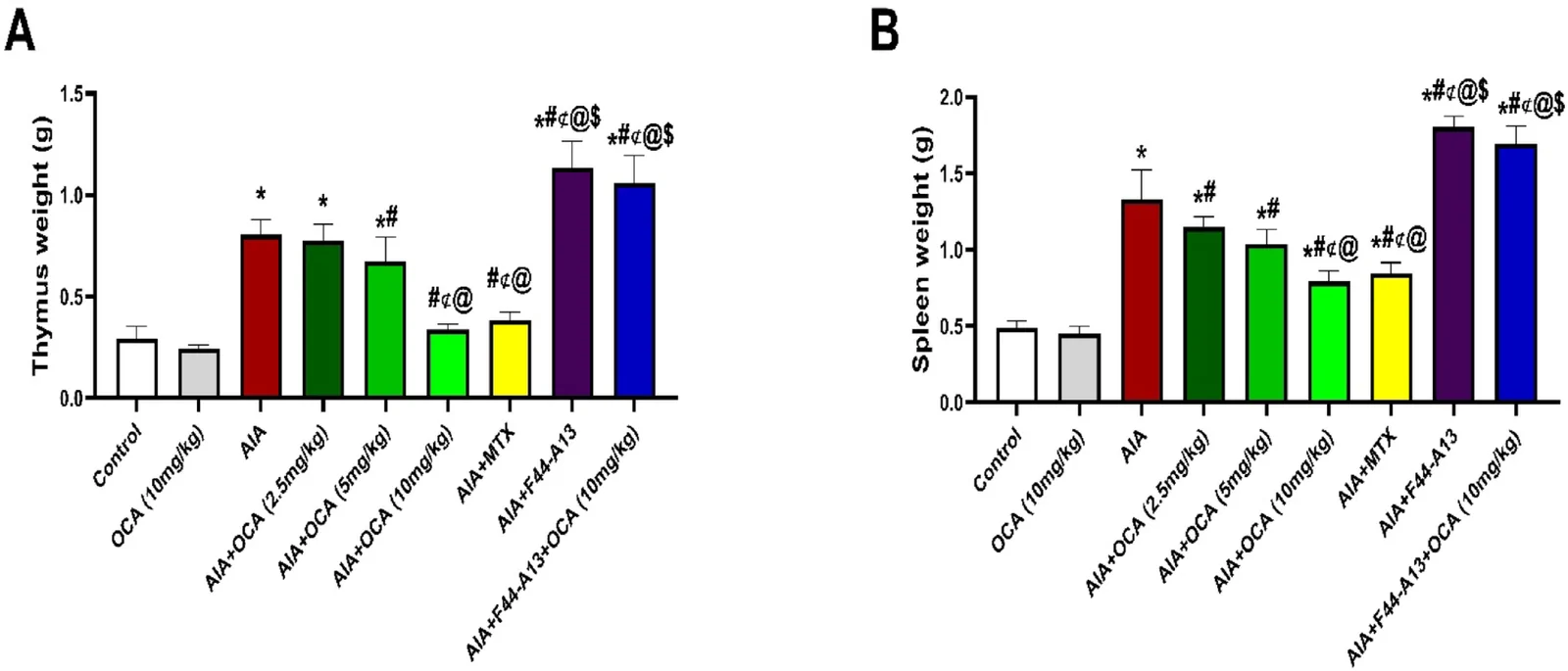
Effect of OCA on CFA-induced changes in thymus weight and spleen weight. Data are expressed as mean ± SD of 12 rats per group, using one-way ANOVA followed by Tukey's post hoc test; F (8.99) = 174.9, F (8.99) = 292; *p* < 0.0001. * vs control, # vs AIA, ȼ vs OCA (2.5mg/kg), @ vs OCA (5mg/kg), $ vs OCA (10mg/kg); OCA: obeticholic acid; AIA: adjuvant-induced arthritis; MTX: methotrexate

The thymus and spleen are sensitive indicators of immunotoxicity, immune modulation, and disease severity in animal models; thus, both organs were weighed and evaluated. One-way ANOVA demonstrated significant differences among groups for both thymus, (F (8.99) = 174.9, *p* < 0.0001) and spleen (F (8.99) = 292, *p* < 0.0001) weights. In the AIA group, administration of CFA significantly increased both the thymus and spleen weights by 2.7-fold each (*p* < 0.0001 vs. control). Coupled with more profound elevation by 1.4-fold in thymus weight and 1.36-fold in spleen weight, when combined with F44-A13 (*p* < 0.0001 vs. AIA). Dose (2.5 mg/kg) didn’t show significant difference compared to AIA group. Meanwhile, dose (5 mg/kg) and dose (10 mg/kg) OCA significantly declined thymus weights by 16% and 58%, as well as spleen weight by 22% and 40% respectively (*p* < 0.0001 vs. AIA). However, Co-administration of F44-A13 markedly reversed the effect of OCA in AIA rats on both thymus and spleen weights Fig. 5.

### Effect of obeticholic acid on serological parameters in CFA-induced arthritic rats

**Fig. 6:**
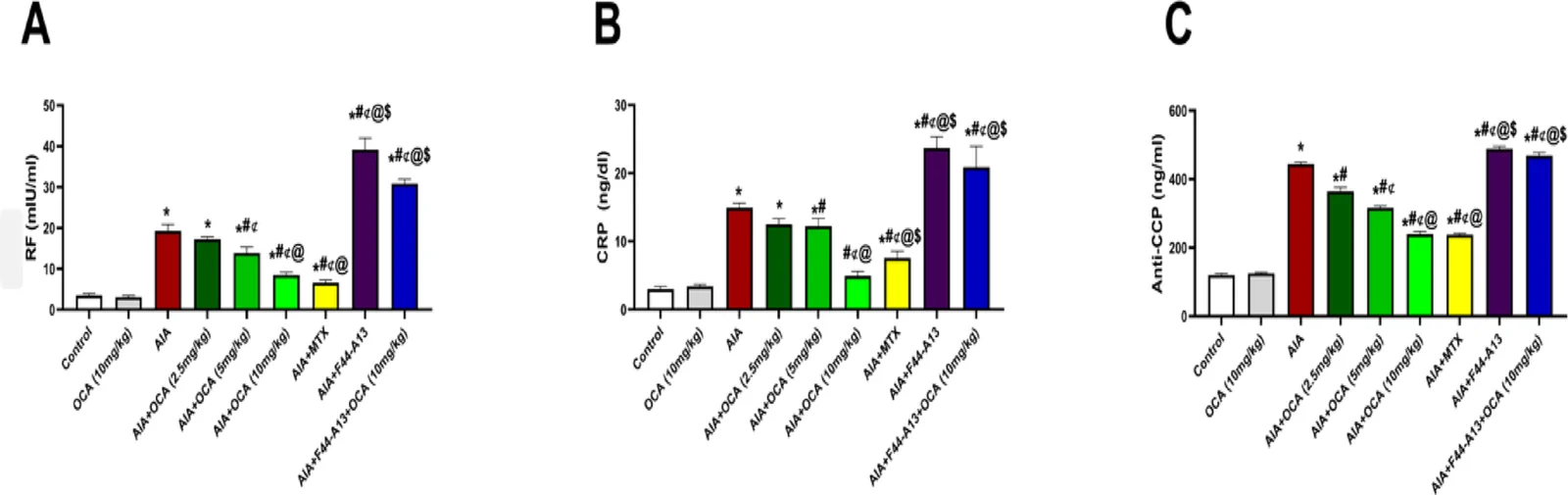
Effect of OCA on CFA-induced alterations in serological parameters (A) RF, (B) CRP, (C) Anti-CCP. Data are presented as mean ± SD of 6 rats per group, using one-way ANOVA followed by Tukey's post hoc test; F (8.45) = 536.1, F (8.45) = 183.8, F (8.45) = 2430; *p* < 0.0001. * vs control, # vs AIA, ȼ vs OCA (2.5mg/kg), @ vs OCA (5mg/kg), $ vs OCA (10mg/kg); OCA: obeticholic acid; AIA: adjuvant-induced arthritis; MTX: methotrexate

Serum markers of systemic inflammation and antibody production were measured and analyzed. One-way ANOVA demonstrated significant differences among groups for the measured parameters: RF (F (8.45) = 536.1, *p* < 0.0001), CRP (F (8.45) = 183.8, *p* < 0.0001), and anti-CCP (F (8.45) = 2430, *p* < 0.0001). In the AIA group, injection of CFA markedly increased the serum levels of RF by 5.5-fold, CRP by 5-fold, and anti-CCP by 3.7-fold (*p* < 0.0001 vs. control). Along with more evident uprise in the preceding parameters when combined with F44-A13, delineated by 2-, 1.6-, and 1.1-fold increase in RF, CRP, and anti-CCP, respectively (*p* < 0.0001 vs. AIA). Dose (2.5 mg/kg) didn’t show significant difference compared to AIA group. On the other hand, treatment with low-dose (5 mg/kg) and high-dose (10 mg/kg) OCA resulted in a noticeable reduction in serum levels of RF by 28%, and 56%, CRP by 18%, and 67%, anti-CCP by 29% and 46%, respectively (*p* < 0.0001 vs. AIA). These effects were blunted by co-administration of F44-A13 with OCA in AIA rats Fig. 6.

### Effect of obeticholic acid on FXR, TGF-β, TGF-βR1, MKK3, TAK1, and p38 in CFA-induced arthritic rats

**Fig. 7:**
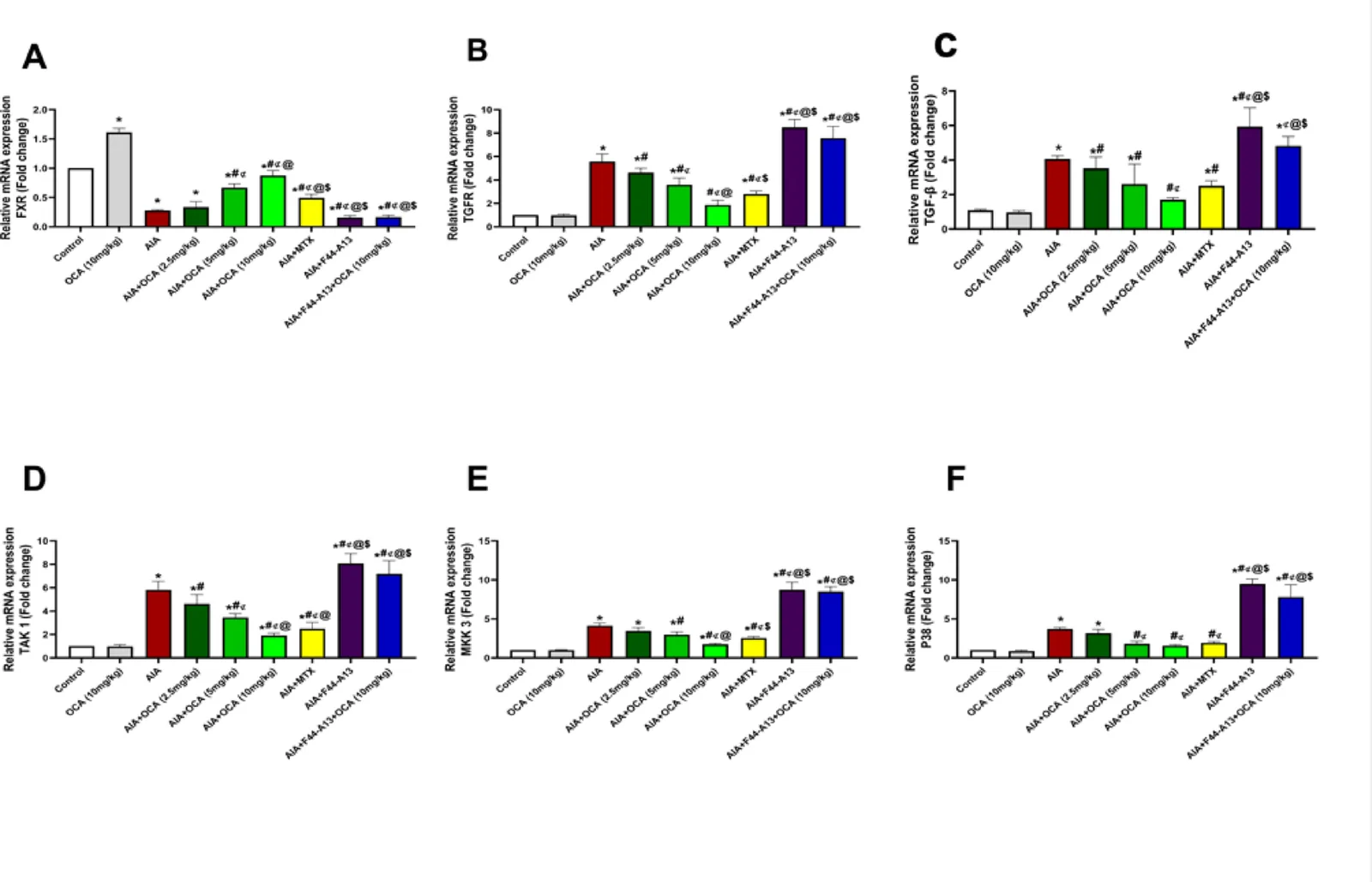
Effect of OCA on CFA-induced changes in the mRNA expression of the studied immune parameters (A) FXR, (B) TGF-β, (C), TGF-βR1, (D) TAK1, (E) MKK3, (F) p38 MAPK. Data are presented as mean ± SD of 6 rats per group, using one-way ANOVA followed by Tukey's post hoc test; F (8.45) = 382.9, F (8.45) = 45.5, F (8,45) = 196.3, F (8,45) = 362, F (8.45) = 345.1, F (8,45) = 194.4; *p* < 0.0001. * vs control, # vs AIA, ȼ vs OCA (2.5mg/kg), @ vs OCA (5mg/kg), $ vs OCA (10mg/kg); OCA: obeticholic acid; AIA: adjuvant-induced arthritis; MTX: methotrexate

To assess the protective mechanism of OCA, the gene expression of the immune regulator nuclear receptor, FXR, and its downstream signaling cascade was measured and analyzed. One-way ANOVA confirmed a significant difference among groups for the expression levels of FXR (F (8.45) = 382.9, *p* < 0.0001), TGF-β (F (8.45) = 45.5, *p* < 0.0001), TGF-βR1 (F (8,45) = 196.3, *p* < 0.0001), MKK3 (F (8.45) = 345.1, *p* < 0.0001), TAK1 (F (8,45) = 362, *p* < 0.0001) and p38 MAPK (F (8,45) = 194.4, *p* < 0.0001). Administration of CFA in the AIA group resulted in a noticeable reduction in FXR expression, along with significant upregulation of TGF-β, TGF-βR1, MKK3, TAK1, and p38 MAPK compared to control rats. Dose (2.5 mg/kg) didn’t show significant difference compared to AIA group. In contrast, treatment with low-dose (5 mg/kg) and high-dose (10 mg/kg) OCA substantially reversed the downregulation of FXR gene expression compared with the insult. Additionally, OCA treatment effectively reduced gene expression of TGF-β, TGF-βR1, MKK3, TAK1, and p38 MAPK compared with the disease group. Simultaneous administration of F44-A13 with OCA largely reversed the changes in the aforementioned parameters (Fig. 7).

### Effect of obeticholic acid on inflammation in CFA-induced arthritic rats

**Fig. 8:**
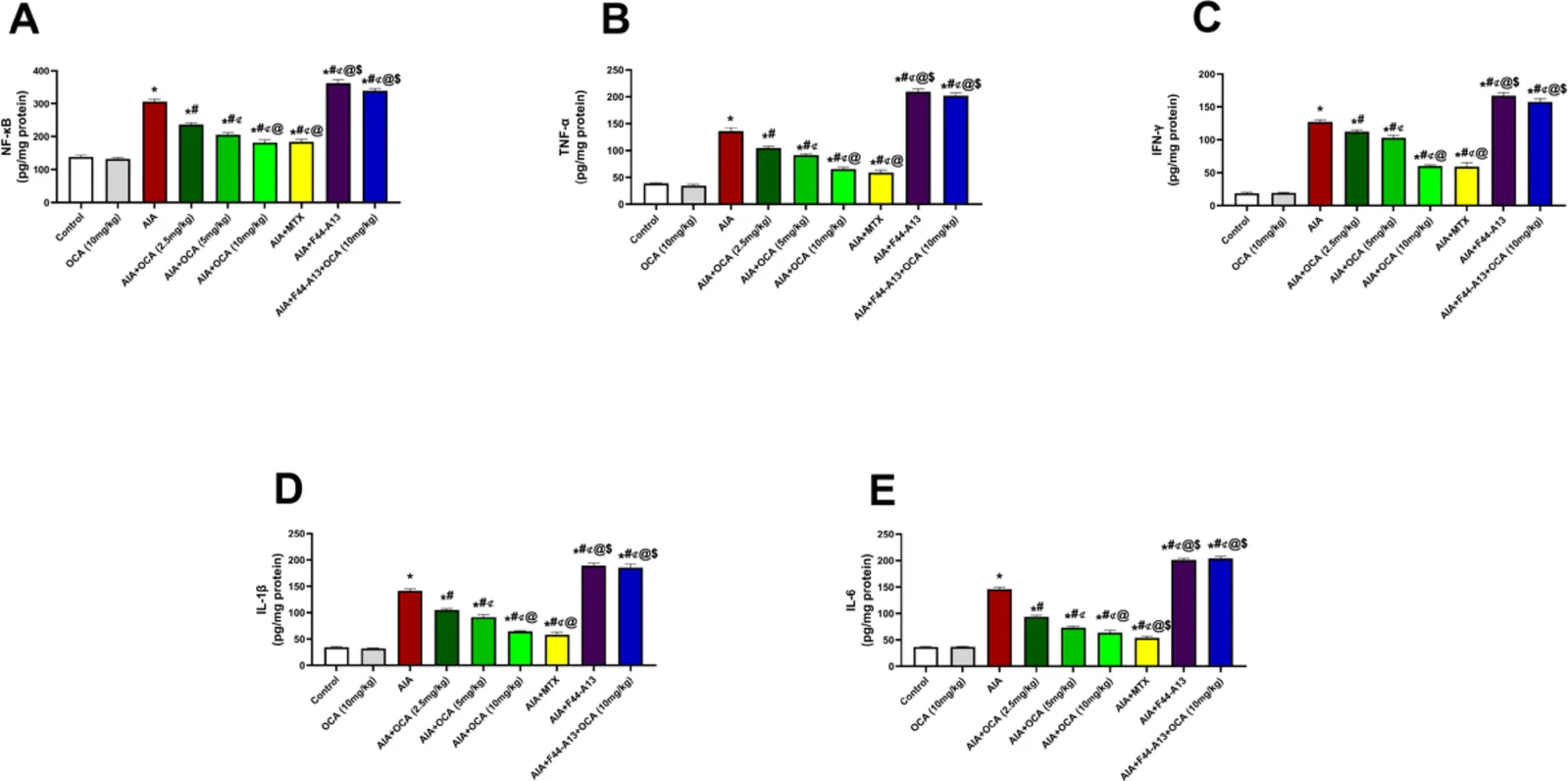
Effect of OCA on CFA-induced alterations in various inflammatory parameters **A** NF-κB p65, **B** TNF-α, **C** IFN-γ, **D** IL-1β, **E** IL-6. Data are presented as mean ± SD of 6 rats per group, using one-way ANOVA followed by Tukey's post hoc test; F (8.45) = 815.4, F (8.45) = 1498, F (8.45) = 1367, F (8.45) = 1408, F (8.45) = 2785; *p* < 0.0001. * vs control, # vs AIA, ȼ vs OCA (2.5mg/kg), @ vs OCA (5mg/kg), $ vs OCA (10mg/kg); OCA: obeticholic acid; AIA: adjuvant-induced arthritis; MTX: methotrexate

The administration of CFA modulates the inflammatory master regulator NF-kB p65 and, therefore, pro-inflammatory cytokine levels, all of which were measured and evaluated. One-way ANOVA showed significant difference between groups for the protein levels of NF-κB p65 (F (8.45) = 815.4, *p* < 0.0001), TNF-α (F (8.45) = 1498, *p* < 0.0001), IFN-γ (F F (8.45) = 1367, *p* < 0.0001), IL-1β (F (8.45) = 1408, *p* < 0.0001), and IL-6 (F (8.45) = 2785, *p* < 0.0001). Administration of CFA in the AIA group showed a significant increase in the levels of NF-κB p65, TNF-α, IFN-γ, IL-1β, and IL-6 by 2.2-, 3.6-, 6.9-, 4.2-, and 4-fold, respectively (*p* < 0.0001 vs. control). Coupled with a greater elevation when co-administered with F44-A13, as shown by an increase by 1.2-, 1.5-, 1.3-, 1.3-, and 1.4-fold, respectively (*p* < 0.0001 vs. AIA). Dose (2.5 mg/kg) didn’t show significant difference compared to AIA group. Conversely, treatment with low-dose (5 mg/kg) and high-dose (10 mg/kg) OCA obviously diminished the levels of NF-kB p65 by 32.7%, and 40.5%, TNF-α by 32.8%, and 52%, IFN-γ by 19.3%, and 53%, IL-1β by 35.4%, and 55% and IL-6 by 50%, and 56.5%, respectively (*p* < 0.0001 vs. AIA). On the contrary, concomitant administration of F44-A13 with OCA blunted the changes in the aforementioned parameters Fig. 8.

### Effect of obeticholic acid on oxidative stress in CFA-induced arthritic rats

**Fig. 9:**
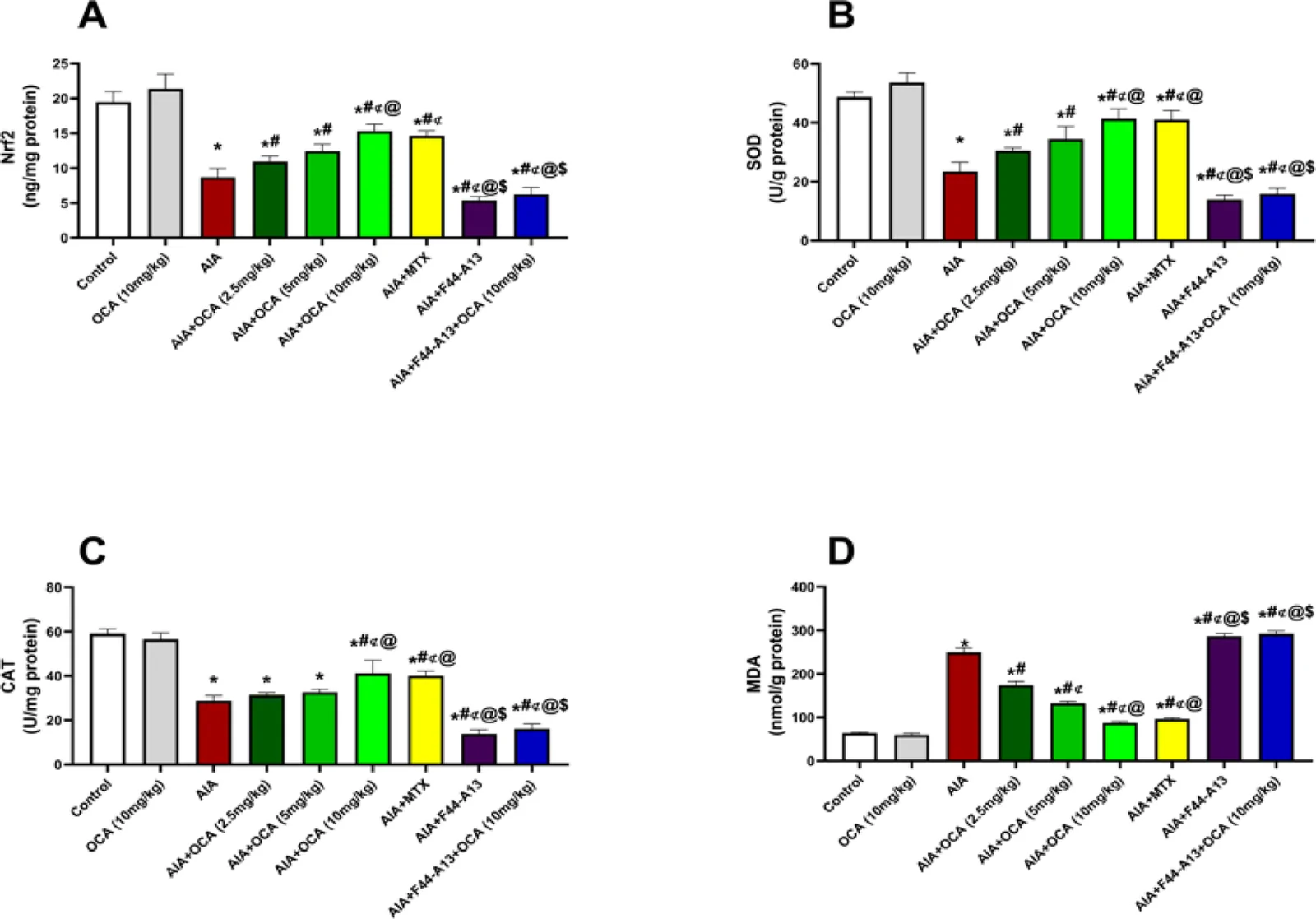
Effect of OCA on CFA-induced abnormalities in oxidative stress parameters (A) Nrf2, (B) SOD, (C) CAT, (D) MDA. Data are presented as mean ± SD of 6 rats per group, using one-way ANOVA followed by Tukey's post hoc test; F (8.45) = 133.1, F (8.45) = 151.2, F (8.45) = 190.7, F (8.45) = 1646; *p* < 0.0001. * vs control, # vs AIA, ȼ vs OCA (2.5mg/kg), @ vs OCA (5mg/kg), $ vs OCA (10mg/kg); OCA: obeticholic acid; AIA: adjuvant-induced arthritis; MTX: methotrexate

Oxidative stress acts as a key factor in the progression of RA. Oxidative stress is implicated in the CFA-induced molecular abnormalities as revealed herein. One-way ANOVA revealed a clear difference between groups for the content of MDA (F (8.45) = 1646, *p* < 0.0001), Nrf2 (F (8.45) = 133.1, *p* < 0.0001), SOD (F (8.45) = 151.2, *p* < 0.0001), and CAT (F (8.45) = 190.7, *p* < 0.0001). CFA injection in the AIA group showed a noteworthy rise in the content of MDA by 3.9-fold, along with a reduction in the content of Nrf2 by 55%, SOD by 52%, and CAT by 51% (*p* < 0.0001 vs. control). Accompanied with more pronounced effect when concurrently given with F44-A13, as revealed by an increase in the content of MDA by 1.15-fold, and a reduction in the content of Nrf2 by 38.2%, SOD by 40.6%, and CAT by 52.1% (*p* < 0.0001 vs. AIA). Dose (2.5 mg/kg) didn’t show significant difference compared to AIA group. While, treatment with low-dose (5 mg/kg) and high-dose (10 mg/kg) OCA resulted in a significant reduction in the content of MDA by 47%, and 65%, together with an increment in the content of Nrf2 by 43%, and 76%, SOD by 47% and 77%, respectively, and only high-dose OCA showed significant increment in the activity of CAT by 43% (*p* < 0.0001 vs. AIA). The antioxidant effects of OCA were markedly diminished when F44-A13 was concurrently administered with OCA in AIA rats Fig. 9.

### Effect of obeticholic acid on the histopathological abnormalities of joints in CFA-induced arthritic rats

**Fig. 10:**
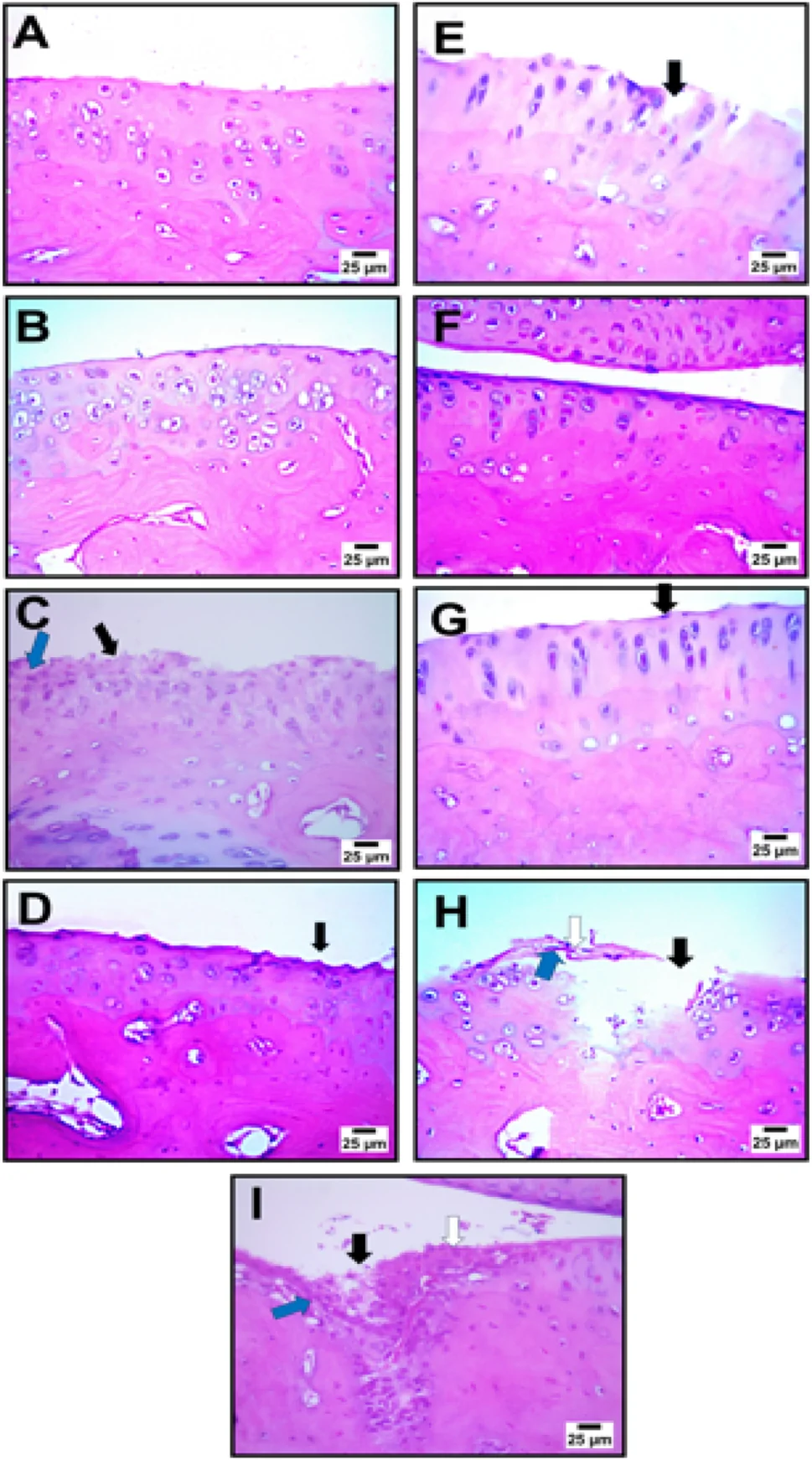
Effect of OCA on CFA-induced histopathological abnormalities. A-I photomicrographs represent the staining of the hind paw with H&E (Scale bar 25). **A** Control group, **B** OCA (10mg/kg)-treated group, **C** AIA group, **D** AIA + OCA (2.5mg/kg)-treated group, **E** AIA + OCA (5mg/kg)-treated group, **F** AIA + OCA (10mg/kg)-treated group **G** AIA + MTX-treated group, **H** AIA + F44-A13-treated group, and **I** AIA + F44-A13 + OCA (10mg/kg)-treated group. Articular surface (black arrow), synovial thickening (white arrow), and inflammatory cells infiltrate (blue arrow). OCA: obeticholic acid; AIA: adjuvant-induced arthritis; MTX: methotrexate

**Fig. 11:**
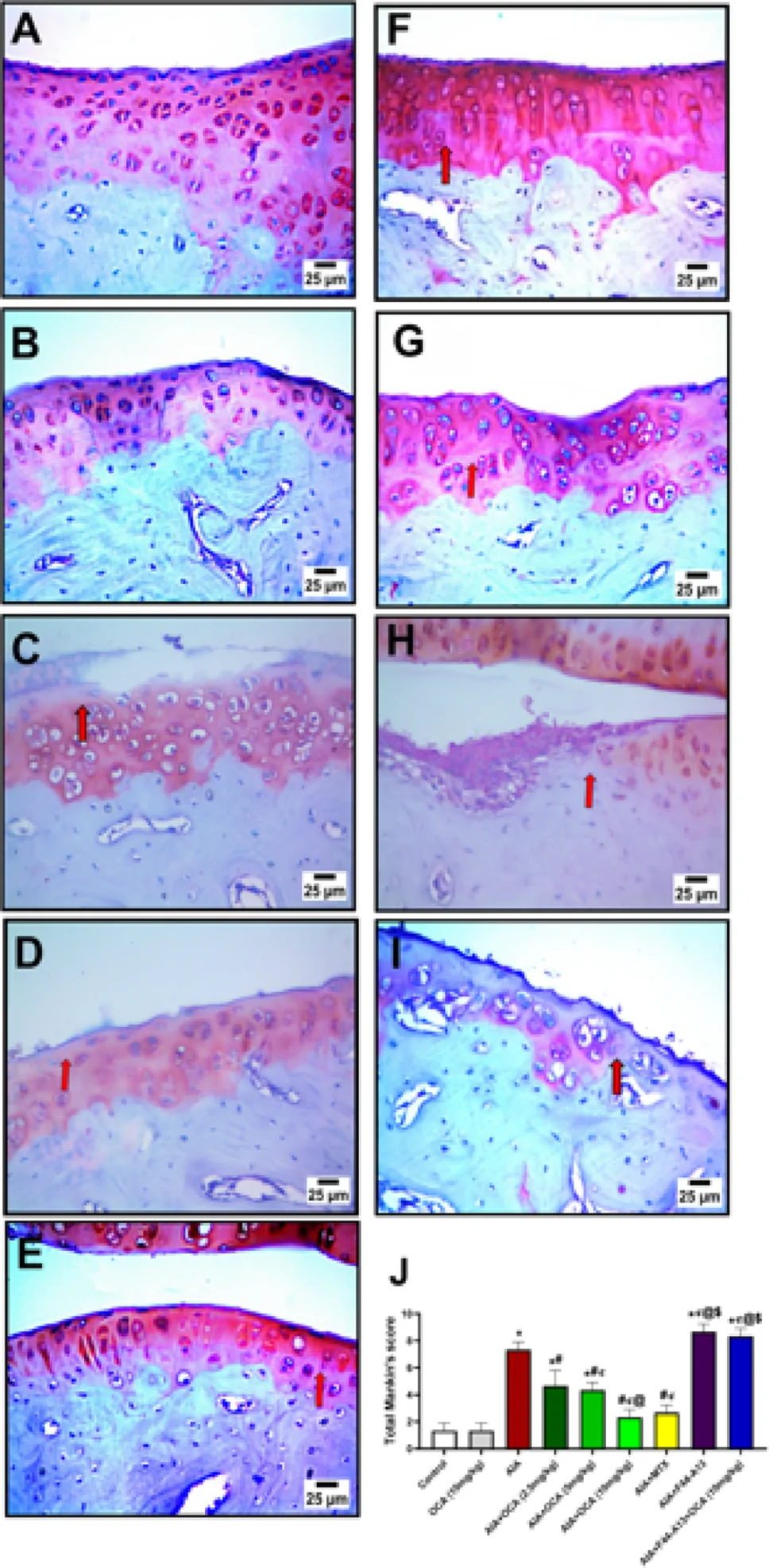
Effect of OCA on CFA-induced histopathological abnormalities. A-J photomicrographs of safranin-O stain of hind paw (Scale bar 25). **A** Control group, **B** OCA (10mg/kg)-treated group, **C** AIA group, **D** AIA + OCA (2.5mg/kg)-treated group, **E** AIA + OCA (5mg/kg)-treated group, **F** AIA + OCA (10mg/kg)-treated group **G** AIA + MTX-treated group, **H** AIA + F44-A13-treated group, **I** AIA + F44-A13 + OCA (10mg/kg)-treated group and **J** Total Mankin's score. Loss of proteoglycan content (red arrow). Data are presented as mean ± SD of 3 rats per group, using one-way ANOVA followed by Tukey's post hoc test; F (8.18) = 57.56; *p* < 0.0001. * vs control, # vs AIA, ȼ vs OCA (2.5mg/kg), @ vs OCA (5mg/kg), $ vs OCA (10mg/kg); OCA: obeticholic acid; AIA: adjuvant-induced arthritis; MTX: methotrexate

The representative photomicrographs taken from the rat hind paw are shown in Figs. 10 and 11. The control rats show no histopathological changes on H&E staining and maintain standard proteoglycan content in the articular surface, as demonstrated by safranin-O staining. On the other hand, CFA administration resulted in severe destruction of the articular surface (black arrow), synovial hyperplasia (white arrow), an inflammatory cell infiltrate (blue arrow), and severe loss of proteoglycan content (red arrow). Interestingly, OCA-treated rats showed significant enhancement and protective efficacy, with an improved articular surface exhibiting mild roughness (black arrow) and mild loss of proteoglycan content (red arrow) in both doses (5 and 10 mg/kg/day), compared to arthritic rats. On the contrary, co-administration of F44-A13 with OCA resulted in marked synovial hyperplasia (white arrow), inflammatory cell infiltrate (blue arrow), destruction of articular surface (black arrow), as well as severe loss of proteoglycan content (red arrow).

Additionally, using Mankin's score to quantify the degree of cartilage damage, our findings show that CFA administration produced a markedly higher score than that of the control group. At the same time, the OCA-treated rats' score was considerably lower than that of AIA rats. On the other hand, co-administration of F44-A13 with OCA attenuated the OCA-associated reduction in cartilage damage. These results suggest that OCA treatment can improve the pathological damage of articular cartilage in CFA-induced arthritic rats.

## Discussion

The current study aimed to evaluated the effects of OCA in CFA-induced arthritis in rats and to provide new insights into the mechanistic pathways underlying these effects. Most investigations exploring the impact of bile acid agonists have focused on conditions such as liver and intestinal inflammation, atherosclerosis, acute lung injury, and multiple sclerosis; however, limited research has examined their potential role in rheumatic disorders (Park and Ji 2017). Although some Previous reports indicated that bile acid agonists can mitigate experimental arthritis by inhibiting inflammatory pathways, the role of FXR in mediating these effects remains largely unexplored (Liu et al. 2011; Li et al. 2019a).

Our study highlights the complex interplay between nuclear receptor activity and immune-inflammatory responses, with a focus on the FXR- associated modulation of inflammatory processes. In clinical practice, anti-inflammatory drugs are recognized as the most widely utilized and effective treatment options (Unterberger 2021). Our findings indicate that OCA treatment was associated with improvements in inflammatory and oxidative stress-related outcomes, as evidenced by a range of observations, including the attenuation of clinical signs and inflammation, accompanied by reduced erythema, pain, edema, and spleen and thymus weight. Moreover, the serum levels of RF, anti-CCP, and CRP were reduced. At the molecular level, OCA treatment was associated with restoration of FXR expression and downregulation of TGF-β/TAK1/MKK3/p38 MAPK-related gene expression, accompanied by suppression of inflammatory mediators and enhancement of Nrf2-associated antioxidant defenses.

The administration of CFA is widely used as an immunological method to induce experimental RA in animal models; it triggers a robust and persistent immune response, leading to infiltration of inflammatory cells, the production of pro-inflammatory cytokines, and, subsequently, joint inflammation. This results in polyarthritis characterized by synovial hyperplasia, cartilage destruction, and bone erosion, closely resembling the pathological features of human RA (Oliveira et al. 2007). The CFA model is highly reproducible and is used to study both acute and chronic phases of arthritis (Oliveira et al. 2007). Despite the inherent limitations of all animal models of disease, those of arthritis show sufficient mimicry of human RA, allowing for greater understanding of the disease and bridging preclinical findings to clinical applications (McNamee et al. 2015). Our results indicated that injection of CFA resulted in exacerbation of clinical manifestations as evidenced by increased thickness, erythema, edema of the hind paw, significant weight loss, and increased spleen and thymus weight. Additionally, the serum levels of RF, anti-CCP, and CRP were elevated, indicating systemic inflammation and antibody production. At the molecular level, the arthritic model group exhibited downregulation of FXR and enhanced mRNA expression of the TGF-β/TAK1/MKK3/p38 MAPK signaling axis, which is correlated with elevated levels of inflammatory markers and a predominance of oxidative stress.

The spleen and thymus weight are commonly measured as indicators of immune system status and modulation in experimental models of RA. Because peripheral and central immune organs are part of the peripheral and central immune organs, respectively, their weight is used to assess the immune-modulatory effects of drugs targeting RA (Wang et al. 2024). Augmented spleen and thymus weight reflect systemic immune activation and ongoing inflammation. Regarding the findings herein, arthritic rats exhibited elevated spleen and thymus weight, whereas treatment with OCA or MTX markedly attenuated their weight. These findings are consistent with an anti-arthritic effect of OCA and were accompanied by reduced indices of systemic immune activation, similar to what was found in a previous study of liver disease (Verbeke et al. 2015).

The farnesoid X receptor, a ligand-activated nuclear receptor primarily recognized for its roles in bile acid, lipid, and glucose metabolism, has recently emerged as an essential immunoregulatory factor in inflammatory diseases, including bone arthritis (Yang et al. 2025). Beyond its metabolic functions, FXR exerts anti-inflammatory effects by regulating transcription of genes involved in cytokine production, oxidative stress responses, and immune cell activation (Lian et al. 2012; Zhang et al. 2017). The bile acid-microbiota-immune axis is a central focus in RA pathophysiology (Xu et al. 2022). Intestinal microbes convert primary bile acids synthesized in the liver into secondary bile acids; consequently, dysbiosis alters this metabolic pathway, leading to shifts in bile acid pool composition (Zhao et al. 2023). It was previously reported that changes in circulating and fecal bile acids in RA predominantly reduced secondary bile acids, which typically have an anti-inflammatory effect, mediated via several nuclear receptors, including FXR (Zhao et al. 2023). Moreover, experimental RA, involving bone destruction and joint swelling, was attenuated by bile acid administration, mediated by downregulation of pro-inflammatory cytokines and shifting the cytokine profile toward an anti-inflammatory state (Liu et al. 2011). Further elucidated, inflammatory cytokines negatively impact osteogenesis by activating osteoclastogenesis while simultaneously suppressing osteoblast differentiation and bone formation. This imbalance leads to progressive bone erosion, a pathological feature of RA (Bingham 2002). Knockout of FXR in animal studies resulted in a defect in bone metabolism, i.e., bone formation and development (Cho et al. 2013; Dong et al. 2025). Our findings are in agreement with the preceding data; CFA administration was associated with reduced FXR mRNA expression and marked joint histopathological abnormalities, whereas OCA treatment was associated with less severe structural cartilage changes. Collectively, these findings are consistent with a potential bone-protective effect of FXR activation in experimental arthritis. Notably, MTX treatment partially restored the FXR expression as compared to the arthritic rats, which may be correlated with reduced inflammatory cytokines, as reported herein, that are documented repressors of FXR transcription (Gadaleta et al. 2011b).

Transforming growth factor-β (TGF-β) is a multifunctional cytokine belonging to the TGF-β superfamily, which regulates immune homeostasis, inflammation, and tissue remodeling. In animal models of RA, TGF-β functions as a double-edged regulator: essential for maintaining immune tolerance yet contributing to chronic inflammation and fibrosis when dysregulated (Gonzalo-Gil and Galindo-Izquierdo 2014). Accumulated evidence from experimental RA studies with comparable 3–4 weeks timelines has suggested that administering 100 µl CFA to male Wistar rats increased TGF-βR expression, findings parallel to those herein (Chen et al. 2024; Belal et al. 2023; Mohaddes et al. 2025). The crosstalk between the FXR and TGF-β signaling represents a critical intersection between metabolic regulation and fibrotic/inflammatory pathways. FXR has been shown to negatively regulate TGF-β- induced profibrotic and proinflammatory responses (Li et al. 2019b). At the molecular level, FXR activation interferes with the TGF-β/SMAD and TGF-β/TAK1 signaling pathways, thereby attenuating downstream phosphorylation events that drive fibroblast activation, extracellular matrix deposition, and the production of inflammatory cytokines (Li et al. 2020; Carino et al. 2018). Moreover, FXR agonists suppress TGF-β-mediated activation of MKK3, p38 MAPK, and NF-κB (Verbeke et al. 2016; Fiorucci et al. 2010; He et al. 2024). In an earlier study, OCA was found to activate FXR and reduce TGF-β protein level, contributing to the mitigation of liver fibrosis (Azizsoltani et al. 2023). Consistent with previous data, our findings indicate that OCA mediates downregulation of TGF-βR mRNA expression while the antagonist findings support FXR involvement. In the same context, the effect of MTX was mediated through the downregulation of TGF-β related gene expression, as reported herein and previously in the AIA model (Abdelmawgoud and Saleh 2018).

TGF-β-activated kinase 1 (TAK1) is a member of the MAPK kinase family. Following TGF-β receptor activation, TAK1 is recruited via the adaptor proteins TAK1-binding protein 1 and 2, often through TNF receptor-associated factor 6-dependent polyubiquitination. This assembly enables autophosphorylation and full activation of TAK1, positioning it as a signaling hub that links TGF-β stimulation to downstream inflammatory and stress-response pathways. The downstream cascades activated by TAK1 involve, at least in part, the MAPK and NF-κB pathways (Fechtner et al. 2017). First, the MAPK cascade encompasses the phosphorylation of MKK-3 to activate p38, which regulates the transcription of genes associated with inflammation, apoptosis, and extracellular matrix remodeling, primarily through transcription factors such as AP-1. Second, the NF-κB pathway is activated via TAK1-induced phosphorylation of the IκB kinase complex, leading to IκB degradation and subsequent NF-κB translocation to the nucleus, which drives the transcription of pro-inflammatory cytokines, adhesion molecules, and matrix metalloproteinases (Ogura and Kondoh 2015). As indicated earlier, arthritic animal models have demonstrated increased protein levels of TAK1, MKK3, and p38 MAPK (Chen et al. 2015). Moreover, pharmacological inhibition of the TAK1-MKK3-p38 MAPK cascade has demonstrated a protective effect against experimental RA in animal models (Chen et al. 2015; Scarneo et al. 2019; Jo et al. 2022; Yang et al. 2018; Rasheed et al. 2010). In light of the data mentioned above, our findings revealed increased mRNA expression of TAK1, MKK3, and p38 MAPK in the arthritic rats. Conversely, the OCA-treated group exhibited downregulation of the aforementioned parameters, an effect may be associated with FXR activation and TGF-β downregulation as previously reported (Li et al. 2020; Ko, et al. 2017). Along the same lines, MTX-treated groups showed downregulation of the p38 MAPK mRNA expression as already stated (Cao et al. 2019). Although MTX is not classically recognized as a direct TAK 1 inhibitor, its upstream modulation of immune cell activation and pro-inflammatory cytokine release, including TNF-α and IL-1β, which are known to activate TAK 1 via receptor signaling, can indirectly attenuate TAK1 signaling or may, at least in part, reflect attenuation of TGF-β-dependent signaling (Hirata et al. 2017).

TGF-β stimulation initiates a signaling cascade that leads to NF-κB activation, primarily via intermediates such as TAK1 (Kim and Choi 2012). NF-κB is a well-established driver of RA pathogenesis, orchestrating the transcription of pro-inflammatory cytokines, promoting oxidative stress, mitochondrial dysfunction, and facilitating bone and cartilage destruction in experimental arthritis models. Inhibiting NF-κB in these models significantly reduces synovial inflammation and joint damage, supporting its therapeutic targeting in RA (Liao et al. 2025). Chief among these pro-inflammatory cytokines are TNF-α, IL-1β, and IL-6, which perpetuate synovial inflammation, joint swelling, pain, and cartilage and bone destruction (Chen et al. 2019; Liu et al. 2021). Elevated TNF-α produced under NF-κB activation stimulates further cytokine and chemokine production, upregulates adhesion molecules, and induces osteoclast differentiation, directly driving bone erosion. IL-1β contributes to matrix degradation and leukocyte recruitment, and IL-6 amplifies B cell activation and autoantibody production, while supporting Th17 T cell responses, further aggravating the autoimmune process (Mateen et al. 2016; Kaneko and Takeuchi 2021).

Additionally, NF-κB participates in signal transduction, activation, and differentiation processes within inflammatory T cells, leading to the production of IFN-γ and IL-17 cytokines that play key roles in maintaining persistent inflammation in rheumatoid synovial tissue (Guo et al. 2024). These observations have prompted interest in FXR-associated modulation of inflammatory pathways as a potential area for further investigation in RA. Notably, OCA has previously been shown to exert potent anti-inflammatory effects by targeting the NF-κB signaling cascade in different animal models of liver injury, kidney injury, placental toxicity, and osteoarthritis (Abdelzaher et al. 2025; Guo et al. 2021). In support of this, similar findings were observed in the present study. This effect may be associated with activation of FXR, leading to suppression of the inflammatory process mainly through NF-κB signaling repression (Hollman et al. 2012). Notably, FXR is expressed in macrophages, and its activation skews polarization toward anti-inflammatory phenotypes (M2) and suppresses NF-κB–driven cytokine production, such as IL-6 and TNF-α (Jaroonwitchawan et al. 2023). Because synovial macrophages are central drivers of inflammation and bone-erosive signals in RA, FXR activity in these cells is a direct mechanistic link to disease modulation (Zheng et al. 2024). In addition, MTX administration resulted in reduced expression of NF-κB and diminished synthesis of cytokines involved in RA pathogenesis such as IL-6, TNF-α, IL-1β and IFN-γ, these outcomes are comparable to those described in earlier investigations (Abdelmawgoud and Saleh 2018; El-Tanbouly and Abdelrahman 2022; Hou et al. 2011).

Recent studies have demonstrated that TGF-β1 signaling may suppress Nrf2-dependent antioxidant gene expression, particularly in models of fibrotic and inflammatory diseases (Bathish et al. 2022; Zhang et al. 2023). This response is credited, at least in part, to sustained activation of NF-κB, which suppresses Nrf2 signaling through competition for transcriptional co-activators or by promoting Keap1 expression (Gao et al. 2022). Moreover, a feedback loop exists, consisting of TGF-Β1-Induced ROS generation, where the generated ROS can activate latent TGF-β1, which in turn produces more ROS, creating a positive feedback loop that amplifies tissue injury and fibrosis (Liu and Desai 2015). A growing body of evidence indicates that ROS actively contribute to RA development, with their levels correlating positively with disease activity due to their ability to damage cellular macromolecules, thereby promoting synovial hyperplasia and joint destruction. Elevated levels of ROS arise primarily from the persistent activation of immune cells, including macrophages, neutrophils, and activated synoviocytes, within the inflamed synovium (Jing et al. 2023). These cells produce ROS as a part of their antimicrobial and signaling function. However, in chronic inflammatory disorders, ROS generation becomes dysregulated, leading to oxidative stress (Ranneh et al. 2017). Nrf2 functions as a critical brake on oxidative stress by upregulating antioxidant enzymes, including CAT and SOD (Ngo and Duennwald 2022). However, this protective mechanism is disrupted by TGF-β1/NF-κB signaling. This observation aligns with our current results, as evidenced by CFA-induced oxidative stress, indicated by elevated levels of MDA, a lipid peroxidation marker, together with reduced Nrf2 and decreased CAT and SOD levels, as previously mentioned (Lal et al. 2021; Hu et al. 2023).

OCA increased Nrf2 content and enhanced antioxidant enzyme levels in a dose-dependent manner, thereby improving antioxidant capacity. These effects may be associated with FXR-related enhancement of Nrf2 signaling and suppression of the TGF-β1/NF-κB axis, consistent with the present findings and previous reports (Wu et al. 2019; Milivojac et al. 2025). However, in RA, it reduces oxidative stress. It enhances antioxidant defenses in inflamed tissues, while simultaneously promoting ROS generation and apoptosis in activated T cells, thereby suppressing pathogenic immune responses (Phillips et al. 2003; Shahin et al. 2011). Although MTX is widely recognized to induce oxidative stress in previous studies of MTX toxicity to lung, liver, kidney, and intestinal tissues (Çetin et al. 2008; Miyazono et al. 2004; Morsy et al. 2013; Ali et al. 2022). However, in the case of RA, it systemically reduces oxidative stress and fosters antioxidant defenses in inflamed tissues, while it can simultaneously promote ROS generation and apoptosis in activated T-cells, thereby suppressing pathogenic immune responses (Phillips et al. 2003; Shahin et al. 2011). Our findings demonstrated the systemic antioxidant property of MTX against CFA-induced oxidative stress, as previously discussed (Zimmerman et al. 2017). The effect may be attributed to the free radical scavenging property and reduced NF-κB expression.

## Conclusion

In conclusion, OCA attenuated CFA-induced arthritis in rats, as reflected by improved clinical, biochemical, oxidative stress, and histopathological outcomes. These effects were associated with restoration of FXR expression, downregulation of TGF-β/TAK1/MKK3/p38 MAPK-related gene expression, reduction in NF-κB p65 and pro-inflammatory cytokine levels, and enhancement of Nrf2-associated antioxidant defenses. The attenuation of OCA effects by F44-A13 supports the involvement of FXR. However, because pathway activation was inferred mainly from expression-level data and because OCA has important clinical safety considerations, further mechanistic and translational studies are required before proposing FXR activation as a therapeutic approach for RA.

## Limitations

Several limitations of the present study should be acknowledged. First, although the CFA-induced arthritis model reproduces several inflammatory, biochemical, and histopathological features of human RA, it does not fully recapitulate the complex autoimmune and chronic nature of the human disease. Second, FXR, TGF-β, TGF-βR1, TAK1, MKK3, and p38 MAPK were primarily assessed at the mRNA level; therefore, changes in gene expression cannot necessarily be interpreted as direct evidence of changes in protein abundance, phosphorylation, or pathway activation. Assessment of phosphorylated TAK1, MKK3, and p38 MAPK, together with additional protein-level analyses, would provide stronger mechanistic confirmation. Third, although the ability of the selective FXR antagonist F44-A13 to attenuate the effects of OCA supports the involvement of FXR, pharmacological antagonism alone cannot establish that all observed effects are exclusively FXR-dependent, and future studies using genetic approaches would strengthen this conclusion. In addition, the study was performed exclusively in male rats over a relatively short experimental period; therefore, sex-dependent responses, long-term efficacy, and potential chronic adverse effects of OCA were not evaluated. Consequently, the present findings should be considered preclinical and require further mechanistic and translational validation before their relevance to the treatment of human RA can be established.

## Data Availability

Data will be made available on request.

## Abbreviations

RA: Rheumatoid arthritis
OCA: Obeticholic acid
CFA: Complete Freund’s adjuvant
AIA: Adjuvant-induced arthritis
FXR: Farnesoid X receptor
TGFR: Transforming growth factor-β receptor
IL-6: Interleukin-6
IL-1β: Interleukin-1 beta
TNF-α: Tumor necrosis factor-alpha
IFN-γ: Interferon-gamma
NF-κB: Nuclear factor kappa B
TAK1: Transforming growth factor-β- activated kinase 1
MKK3: Mitogen-activated protein kinase kinase 3
p38 MAPK: P38 mitogen-activated protein kinase
SOD: Superoxide dismutase
CAT: Catalase
MDA: Malondialdehyde
Nrf2: Nuclear factor erythroid 2–related factor 2
CRP: C-reactive protein
RF: Rheumatoid factor
Anti-CCP: Anti-cyclic citrullinated peptide
IKK: IκB kinase
Keap1: Kelch-like ECH-associated protein 1
ROS: Reactive oxygen species
NSAIDs: Non-steroidal anti-inflammatory drugs
csDMARDs: Conventional synthetic disease-modifying anti-rheumatic drugs
H &E: Hematoxylin and Eosin
